# An arrayed CRISPR screen of primary B cells reveals the essential elements of the antibody secretion pathway

**DOI:** 10.3389/fimmu.2023.1089243

**Published:** 2023-02-13

**Authors:** Stephanie Trezise, Isabella Y. Kong, Edwin D. Hawkins, Marco J. Herold, Simon N. Willis, Stephen L. Nutt

**Affiliations:** ^1^ Walter and Eliza Hall Institute of Medical Research, Parkville, VIC, Australia; ^2^ Department of Medical Biology, The University of Melbourne, Parkville, VIC, Australia; ^3^ Center for Immunology and Inflammatory Diseases, Massachusetts General Hospital, Harvard Medical School, Harvard University, Boston, MA, United States; ^4^ Department of Pediatrics, Division of Pediatric Hematology/Oncology, Weill Cornell Medicine, New York, NY, United States

**Keywords:** plasma cell, immunodeficiency, humoral immunity, *in vitro* differentiation, endoplasmic reticulum, unfolded protein response, ER associated degradation (ERAD)

## Abstract

**Background:**

Humoral immunity depends on the differentiation of B cells into antibody secreting cells (ASCs). Excess or inappropriate ASC differentiation can lead to antibody-mediated autoimmune diseases, while impaired differentiation results in immunodeficiency.

**Methods:**

We have used CRISPR/Cas9 technology in primary B cells to screen for regulators of terminal differentiation and antibody production.

**Results:**

We identified several new positive (*Sec61a1*, *Hspa5*) and negative (*Arhgef18*, *Pold1*, *Pax5*, *Ets1*) regulators that impacted on the differentiation process. Other genes limited the proliferative capacity of activated B cells (*Sumo2*, *Vcp*, *Selk*). The largest number of genes identified in this screen (35) were required for antibody secretion. These included genes involved in endoplasmic reticulum-associated degradation and the unfolded protein response, as well as post-translational protein modifications.

**Discussion:**

The genes identified in this study represent weak links in the antibody-secretion pathway that are potential drug targets for antibody-mediated diseases, as well as candidates for genes whose mutation results in primary immune deficiency.

## Highlights

- Study revealed key dependencies in B-cell terminal differentiation and antibody secretion.
- These genes are potential therapeutic targets for treating antibody-mediated diseases and candidate causative genes for primary antibody deficiencies.

## Introduction

The differentiation of mature B cells into antibody secreting cells (ASCs) is an essential component of the adaptive immune response. The ASC compartment is comprised of short-lived proliferating plasmablasts and long-lived, generally post-mitotic, plasma cells. The antibodies produced by these cells are important for the elimination of pathogens and the persistent secretion of these antibodies after pathogen clearance provides long-term protection against re-infection. Conversely, the inability to efficiently produce antibodies results in immune deficiency. Despite the crucial roles that ASCs play in immune health, we still lack a complete understanding of the factors that regulate their differentiation and antibody secretion.

While many factors have been implicated in driving this terminal differentiation process, most of the focus to date has been on the transcription factors Irf4, Blimp-1 (encoded by *Prdm1*) and Xbp1 (1). Irf4 is essential for the initial stages of the ASC differentiation process, in part due to its role in driving expression of *Prdm1*/Blimp-1 (2–5). Blimp-1, while not required for the initiation of the differentiation process, is essential for the formation of ASCs, as it silences the expression of the genes responsible for maintaining B cell identity including *Pax5* (6–9). In ASCs, Blimp-1 maintains the expression of genes involved in antibody secretion, either through direct activation or through the recruitment of chromatin modifying complexes (9, 10).

ASCs are a highly specialized cell type, devoting approximately 70% of their transcriptome to the synthesis of the *Igh* and *Igl* chains (11). This unique transcriptional program is accompanied by a reorganization of the cellular cytoplasm to allow for the formation of parallel arrays of rough endoplasmic reticulum (ER) that is necessary to facilitate massive antibody secretion. The high rates of antibody synthesis make ASCs extremely sensitive to ER stress and, consequently, they are particularly dependent on ER stress responses such as the ER-associated degradation (ERAD) pathway and the unfolded protein response (UPR) (12). Xbp1 is a key regulator of the UPR that drives increases in cell size and ER content and promotes expression of genes involved in ER homeostasis and secretory protein production (7, 9). Xbp1 is not required for the differentiation or survival of ASCs, however, the UPR activity and secretory capacity of *Xbp1*-deficient ASCs is greatly diminished (9, 13, 14). It is highly likely that there are additional, as yet unknown, genes which are also essential for the generation and function of ASCs.

We have previously performed a comprehensive transcriptional analysis of the terminal differentiation process from naïve B cell through to long-lived bone marrow plasma cells (11). This study revealed that despite differences in anatomical location, lifespan and proliferation status, ASCs share a core transcriptional signature. In addition to known regulators of ASC biology, *Prdm1*, *Irf4* and *Xbp1*, this ASC gene signature contained many genes whose functions have not been previously characterized or have not been examined in the context of ASCs. To interrogate the function of these genes, we have developed a CRISPR-Cas9 mediated arrayed targeted screen in primary mouse B cells, with the ability to measure multiple parameters in parallel, including antibody secretion. We have used this system to identify genes positively and negatively influencing the differentiation, proliferation, survival and secretion capacity of ASCs. Several of the genes identified in these screens as being required for ASC differentiation or antibody secretion have been implicated in primary antibody deficiencies. In most primary antibody deficiency patients, the genetic cause remains undetermined, therefore, the additional hits from these screens represent excellent candidates for the genes that underpin these diseases. Conversely, the genes identified as negative regulators of differentiation may play roles in preventing antibody-mediated autoimmune diseases or allergy.

## Results

### An arrayed targeted CRISPR screen for primary murine B cells

**Figure 1:**
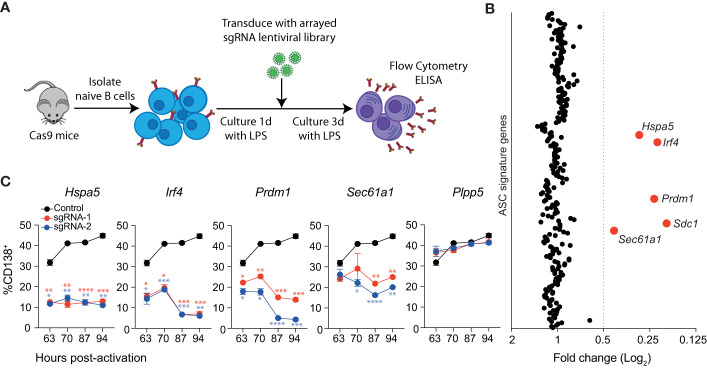
Identification of genes essential for LPS driven B cell differentiation *in vitro*. **(A)** Workflow of targeted CRISPR screen. Naïve splenic B cells were isolated from Cas9 expressing transgenic mice, activated with LPS and transduced with an arrayed lentiviral library that co-expressed specific sgRNAs and BFP. Three days after transduction, cells were analyzed by flow cytometry and culture supernatant by ELISA. **(B)** Average fold changes in the proportion of transduced cells (BFP^+^) that express CD138 for each targeted gene relative to the untransduced control. Genes with a fold change ≤0.5 are labelled and highlighted in red. Data points represent the mean of 2 independent sgRNAs from 2 replicate experiments. **(C)** Proportion of CD138^+^ cells among cells transduced with sgRNAs (BFP^+^) targeting *Hspa5*, *Irf4, Prdm1, Sec61a1* or the *Plpp5* control at the indicated time post-activation with LPS. Data points represent the mean of triplicate wells and error bars indicate the S.E.M. *p < 0.05, **p < 0.01, ***p < 0.001, ****p < 0.0001. Data in **(C)** is representative of 3 independent experiments.

We sought to establish a CRISPR-Cas9 based screening system, which would allow the identification of genes that are essential for the generation, survival and/or antibody-secreting capacity of ASCs. While conventional pooled CRISPR-Cas9 screens can detect genes required for differentiation and survival, they are not able to assess defects in antibody secretion (15, 16). We optimized a 96-well transfection and primary B cell transduction protocol that consistently results in transduction rates above 80% **(** **Supplementary Figures 1A, B** **)**. To test this system, cells were transduced with sgRNAs targeting *Sdc1*, which encodes CD138, a surface marker that serves as a proxy for ASC differentiation. Naïve splenic B cells were isolated from Cas9 transgenic mice and stimulated for 24 hours with LPS before lentiviral transduction with sgRNAs. Following transduction, the cells were returned to culture under LPS stimulation for a further three days before analysis **(** **Figure 1A** **)**. At three days post-transduction, there were few detectable CD138^+^ cells within the sgRNA transduced populations **(** **Supplementary Figure 1C** **)**. To ensure that this system could block the differentiation of B cells, we measured the effect of targeting *Prdm1*, an essential driver of the differentiation process (8), and *Plpp5*, an ASC signature gene that does not influence differentiation (17). At three days post-transduction, cells transduced with sgRNAs targeting *Prdm1* showed an 80-90% decrease in the proportion of differentiated cells **(** **Supplementary Figure 1D** **)**. In contrast, cells transduced with sgRNAs targeting *Plpp5* did not display any difference in the proportion of CD138^+^ cells compared to controls **(** **Supplementary Figure 1D** **)**. To examine the antibody-secreting capacity of the transduced cells, the concentration of IgM in the culture supernatant was measured by ELISA. Cells transduced with sgRNAs targeting *Prdm1* showed a 95% reduction in IgM secretion relative to the control, while *Plpp5* targeting sgRNAs did not impact on antibody secretion rates **(** **Supplementary Figure 1E** **)**. From these data, we conclude that, despite the short timeframe of the assay, this system is suitable for identifying genes that are essential for B cell differentiation and antibody secretion.

### Identification of positive regulators of ASC differentiation

We used this system to interrogate the ASC gene signature to identify novel regulators of ASC differentiation *in vitro*. Of the 301 originally defined ASC signature genes, we screened sgRNAs corresponding to 258 protein-coding genes (**Table S1**). Naïve splenic B cells were transduced with an arrayed lentiviral library containing two sgRNAs against each gene such that each well received a single sgRNA and were cultured as above **(** **Figure 1A** **)**. The impact of each sgRNA on differentiation was determined by examining the proportion of transduced (BFP^+^) cells that expressed CD138. The cut-off for genes of interest was arbitrarily set to sgRNAs that reduced the proportion of CD138^+^ cells by 50% relative to the untransduced controls for each plate **(** **Figure 1B** **)**. In agreement with **Supplementary Figures 1C, D**, sgRNAs targeting *Prdm1* or *Sdc1* resulted in a decrease in CD138^+^ cells. We also observed a reduction in differentiated cells following transduction with sgRNAs targeting *Irf4, Hspa5*, and *Sec61a1*. There was strong agreement between the effect of sgRNA pairs directed against the same gene, and a consistent effect of targeting the same gene across replicate screens **(** **Supplementary Figure 2A** **)**. These results demonstrate that most genes within the ASC signature are not required for differentiation to CD138^+^ ASCs, at least in the context of this *in vitro* assay.

The single timepoint examined in the screen assay does not provide any information as to how these genes are influencing the kinetics of the differentiation process. To investigate this, we repeated the assay, focusing on the genes of interest and including multiple timepoints **(** **Figure 1C** **)**. As expected, targeting *Plpp5* did not have any effect on the frequency of differentiated cells at any examined timepoint, while targeting *Irf4* or *Prdm1* resulted in a significant reduction in differentiated cells at all examined timepoints. Similarly, *Hspa5* targeting resulted in a significantly decreased frequency of CD138^+^ cells at all timepoints. *Hspa5*, encodes Grp78 or BiP, a major regulator of the UPR, which binds to ER stress sensors, keeping them in an inactive state (18). It is likely that the *Hspa5* targeted cells have unrestrained activation of their UPR, resulting in cell death, and that the cells undergoing differentiation and upregulating antibody production would be the most sensitive to this stress. *Sec61a1* encodes the largest subunit of the Sec61 complex, which controls the co-translational or post-translational transport of polypeptides into the ER lumen and peptide insertion into the ER membrane (19, 20). *Sec61a1* targeted cells initially showed similar rates of differentiation to untransduced cells, however, at later timepoints there was a significant decrease in the frequency of differentiated cells. It is of interest that the ASC signature examined in this screen contains 36 other genes that are considered components of the UPR that did not impact on ASC differentiation rates.

### Negative regulators of ASC differentiation

**Figure 2:**
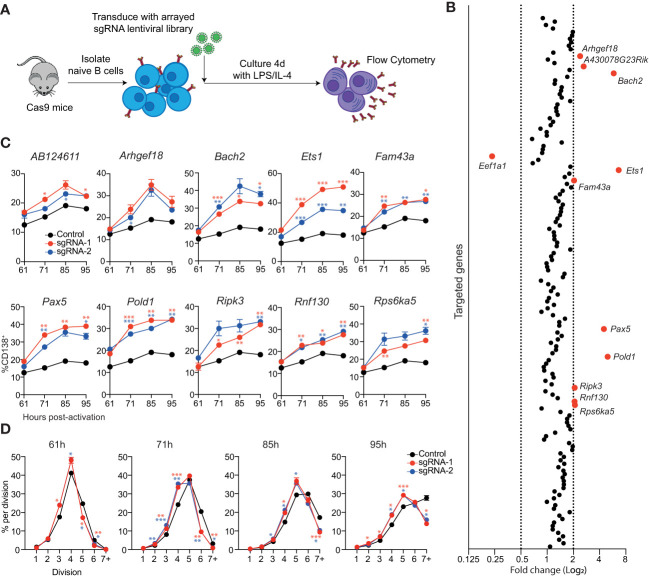
Identification of genes that repress ASC differentiation *in vitro.* **(A)** Overview of experimental workflow for targeted arrayed CRISPR/Cas9 screen. Naïve splenic B cells were isolated from Cas9 expressing transgenic mice and transduced with an arrayed lentiviral sgRNA library. Following transduction, cells were cultured in LPS + IL-4 for 4 days before analysis by flow cytometry. **(B)** Each data point represents the average fold change in the proportion of transduced cells (BFP^+^) that are CD138^+^ for both sgRNAs targeting a particular gene relative to the untransduced controls on the same plate. Genes with a fold change of ≤0.5 or ≥2 are labelled and highlighted in red. Data points represent the mean of 2 independent sgRNAs from 2 replicate experiments. **(C)** Naïve B cells from Cas9 transgenic mice were transduced with sgRNAs targeting the indicated genes and cultured in LPS. At the indicated timepoints, the proportion of CD138^+^ cells within the transduced (BFP^+^) population was assessed by flow cytometry. **(D)** Naïve B cells from Cas9 transgenic mice were labelled with the division tracking dye CTY, transduced with sgRNAs targeting *Pold1* and cultured in LPS. At the indicated timepoints, the dilution of CTY within the transduced (BFP^+^) population was assessed by flow cytometry. Data points represent the mean of triplicate wells and error bars indicate the S.E.M. **(C, D)** are representative of 2 independent experiments. *p < 0.05, **p < 0.01, ***p < 0.001.

We hypothesized that this screening assay, with minor modifications, would also be suitable to identify negative regulators of the B-cell differentiation process **(** **Figure 2A** **)**. In contrast to the screen for positive regulators, the cells were cultured in LPS + IL-4 as this condition induces a relatively weak differentiation response and, therefore, enhanced differentiation rates should be more apparent. We also introduced the sgRNAs into unstimulated B cells, to allow the targeting of these genes early in the differentiation process. To validate this approach, B cells were transduced with sgRNAs directed against *Bach2*, as *Bach2^-/-^* B cells display enhanced differentiation (21) and *BACH2* variants are associated with many autoimmune and allergic diseases (22). The transduction rate of unstimulated B cells, although reduced compared to that of activated B cells, was sufficient for the development of a robust assay **(** **Supplementary Figure 3A** **).** At 4 days post-transduction, *Bach2* sgRNA transduced cells displayed a 4-fold increase in the rate of differentiation compared to the untransduced controls **(** **Supplementary Figure 3B** **)**.

To identify potential negative regulators of ASC differentiation, we reanalyzed the RNAseq data that was used to generate the ASC gene signature and focused on genes that had a 3-fold higher expression in follicular B cell (FoB) samples compared to all ASC subsets (≤0.05 false discovery rate (FDR), ≥32 fragment per kilobase million reads (FPKM) in FoB samples) (11). This strategy generated a candidate list of 155 genes that are downregulated during differentiation that included many canonical B cell genes including, *Cd19*, *Cd22*, *Ms4a1* (*Cd20*), *Bcl6*, *Pax5* and *Ebf1* **(** **Supplementary Figure 3C** and **Table S2** **)**. An arrayed lentiviral library was generated that contained two sgRNAs targeting each gene within this gene list. In agreement with the validation experiments, targeting *Bach2* resulted in an increased proportion of CD138^+^ cells **(** **Figure 2B** **)**. The other sgRNAs that resulted in a large increase in differentiation were directed against *Ets1*, *Pax5* and *Pold1*. We also observed a more modest effect in targeting *AB124611*, *Arhgef18* (or *A430078G23Rik* which is the same gene as *Arhgef18)*, *Fam43a*, *Ripk3*, *Rnf130* and *Rsp6ka5*. There was an additional gene, *Eef1a1*, which encodes a translation elongation factor, that resulted in a decrease in the proportion of CD138^+^ cells.

To analyze the kinetics of differentiation in the targeted cells we performed a time course in cultures supplemented with LPS **(** **Figure 2C** **)** or LPS + IL-4 **(** **Supplementary Figure 3D** **)**. Cells transduced with sgRNAs targeting *AB124611* or *Arhgef18* displayed a slight increase in differentiation in both conditions at all timepoints. All other genes resulted in a significant increase in differentiation at multiple timepoints, with the transcription factors *Bach2*, *Ets1*, *Pax5* and the DNA polymerase *Pold1* targeted cultures having the most pronounced impact. *Pold1* is involved in lagging strand synthesis during DNA replication and G1 to S-phase transition (23, 24) Indeed, we observed that *Pold1* targeted cells display delayed proliferation kinetics **(** **Figure 2D** **)** that may be indirectly driving increased rates of differentiation by slowing cell cycle progression (25, 26). The pathways through which *AB124611* (unknown function), *Arhgef18/A430078G23Rik* (guanine nucleotide exchange factor), *Fam43a* (unknown function), *Ripk3* (necroptosis pathway), *Rnf130* (E3 ubiquitin ligase) and *Rps6ka5* (S6 kinase family) limit ASC differentiation is unclear and requires further investigation.

### Identification of B cell proliferation and survival regulators

**Figure 3:**
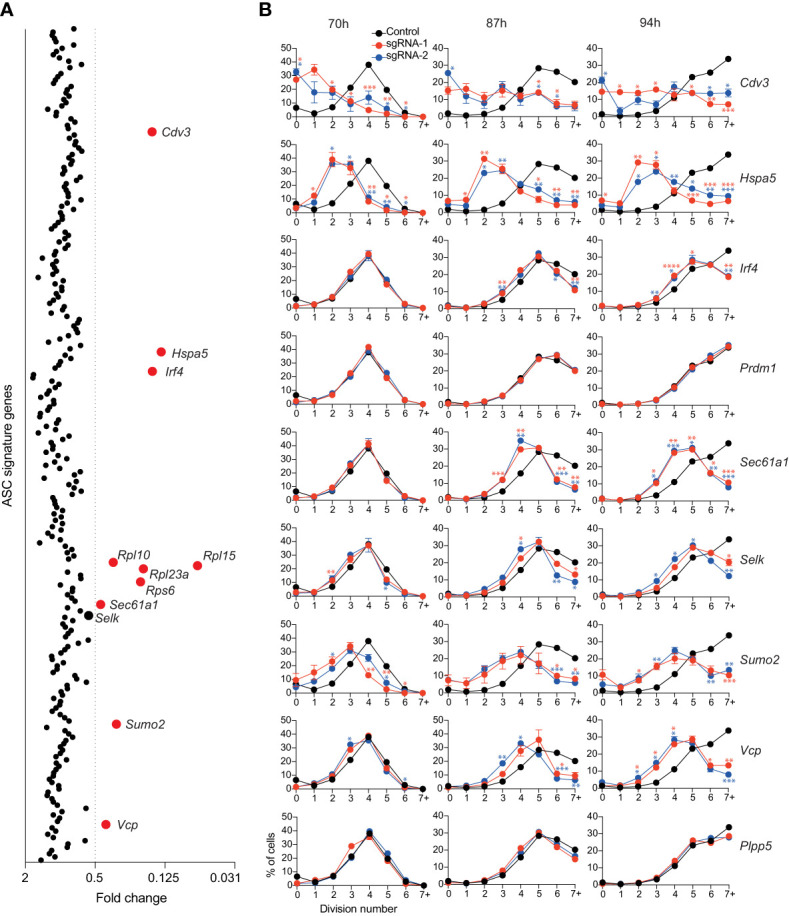
Genes affecting total live cell number. **(A)** Experimental workflow is described in **Figure 1A**. Average fold changes in the total number of live cells for each targeted gene relative to the untransduced control. Genes with a fold change of ≤0.5 are labelled and highlighted in red. Data points represent the mean of 2 independent sgRNAs from 2 replicate experiments. **(B)** Naïve B cells from Cas9 transgenic mice were labelled with the division tracking dye CTY, activated with LPS and transduced with sgRNAs targeting *Irf4*, *Prdm1*, *Cdv3*, *Sumo2*, *Sec61a1*, *Hspa5*, *Selk, Vcp* or the *Plpp5* control. At the indicated timepoints, the dilution of CTY was assessed by flow cytometry. Data points represent the mean proportion of cells in each division from triplicate wells. Error bars indicate the S.E.M. Representative of 3 independent experiments. *p < 0.05, **p < 0.01, ***p < 0.001, ****p < 0.0001.

To investigate potential regulators of B cell proliferation or survival, we reanalyzed the data from the positive regulator screen, this time examining the total live cell number **(** **Figure 3A** **)**. 10 genes which influenced B cell survival and/or proliferation were identified (*Cdv3, Hspa5, Irf4, Rpl10, Rpl15, Rpl23a, Rps6, Sec61a1, Sumo2, Vcp*). Comparison with the differentiation results demonstrated that some of these genes (*Irf4*, *Hspa5* and *Sec61a1*) affected both cell number and differentiation, while the other genes identified only influenced cell number (**Supplementary Figure 2B**). *Irf4* has been linked to cell division in activated B cells as it directly induces the expression of genes involved in proliferation, including *Myc* (27, 28). *Sumo2* has previously been implicated in proliferation and cell survival as Sumo2-deficient mouse embryonic fibroblasts have decreased cell cycling and an increased cell death compared to WT cells (29). All the genes within the ASC gene signature that encode ribosomal proteins (*Rpl10, Rpl15, Rpl23a*, *Rps6*) were identified as having a strong effect on cell number. As efficient protein translation is essential for cell division and survival, it is unsurprising that targeting these genes would have a dramatic effect on cell numbers. As discussed previously, *Hspa5*/Grp78 is a major regulator of ER homeostasis and a reduction in Grp78 concentration can result in cell death (18). *Vcp* and *Selk*, a gene that was just above our fold change cut-off, both encode components of the ERAD pathway, which is responsible for detecting misfolded proteins and targeting them for proteasomal degradation before they can accumulate and trigger the terminal UPR (30–33). Curiously, there are additional genes within the ASC gene signature (*Derl1, Derl3, Edem3, Herpud1, Hsp90b1, Os9, Sel1l*) that encode components of the ERAD pathway which, when inactivated, did not have a clear impact on the total live cell number. This may reflect a redundant role between family members within this pathway.

By measuring cell number, we could not dissect the effects of genes that affected cell survival and genes that affected proliferation. Therefore, to interrogate these processes, Cas9 expressing B cells were labelled with the division tracking dye Cell Trace Yellow (CTY), activated for 24 hours with LPS, transduced with sgRNAs targeting the genes of interest and CTY dilution was assessed at multiple timepoints post-transduction. The genes encoding ribosomal proteins are essential for many basic cellular processes and were therefore excluded from further investigation. B cell division and differentiation are linked processes, with the probability of differentiation increasing with each division, therefore genes that were identified as regulators of differentiation were included even if they did not meet the reduction in cell number threshold (34, 35). We also included *Plpp5*, which does not influence B cell number, as an additional control. The CTY dilution profiles of cells transduced with sgRNAs targeting *Prdm1* and *Plpp5* overlapped with the untransduced controls, indicating that these genes do not influence B cell proliferation **(** **Figure 3B** **)**. It has previously been demonstrated that *Irf4*-deficient B cells have a reduced proliferative capacity in response to LPS compared to WT B cells (27, 36). This proliferative defect was confirmed by our data and was most notable at later timepoints where cells transduced with *Irf4* targeting sgRNAs had stalled in their proliferation **(** **Figure 3B** **)**. Targeting *Cdv3, Hspa5*, *Sumo2* or *Vcp* caused a dramatic reduction in proliferation capacity while targeting *Selk* or *Sec61a1* resulted in a less severe alteration in cell division. Clearly, there are many direct and indirect approaches to target B cell proliferation and thus impact on ASC differentiation and function.

### Identification of antibody secretion regulators

**Figure 4:**
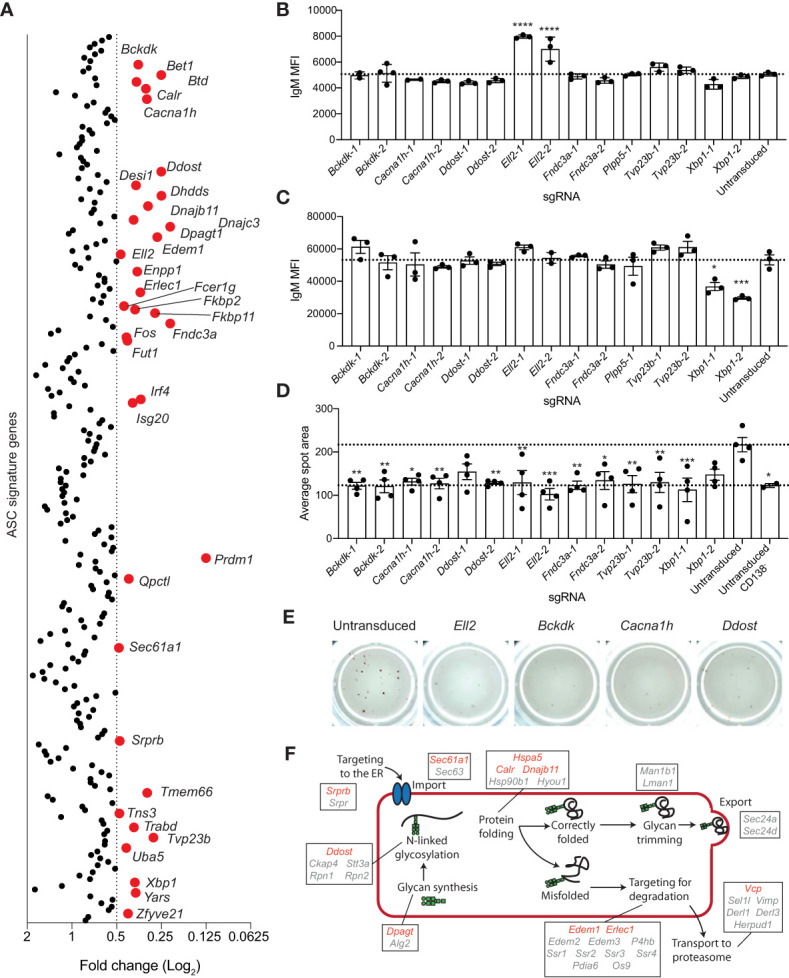
Genes essential for antibody secretion. **(A)** Experimental workflow is described in **Figure 1A**. The concentration of IgM in the culture supernatant was measure by ELISA and normalized to the live cell number. Data are presented as average fold change in IgM per cell for each targeted gene relative to the untransduced control. Genes with a fold change of ≤0.5 are labelled and highlighted in red. Data points represent the mean of 2 independent sgRNAs from 2 replicate experiments. **(B, C)** Naïve B cells from Cas9 transgenic mice were activated with LPS, transduced with sgRNAs targeting *Xbp1*, *Ell2*, *Bckdk*, *Cacna1h*, *Ddost*, *Fndc3a*, *Tvp23b*, or the *Plpp5* control, and cultured for a further 3 days before analysis. Mean fluorescence intensity (MFI) of IgM on the **(B)** plasma membrane or **(C)** total cellular IgM. **(D, E)** At 2 days post-transduction, transduced and untransduced cells were sorted and re-cultured overnight before transfer to ELISpot plates. **(D)** Average spot size formed and **(E)** representative wells are shown. Error bars indicate S.E.M. and dotted lines indicate the mean of the untransduced samples. Data in **(B–E)** are representative of 2-3 independent experiments. **(F)** Overview of the ER protein folding pathway (KEGG pathway: mmu04141) with genes within the ASC gene signature labelled. Red indicates secretion screen hits and grey indicates genes that did not reach the fold change cut-off. *p < 0.05, **p < 0.01, ***p < 0.001, ****p < 0.0001.

An advantage of performing these screens in an arrayed format is that it allows for the identification of genes that regulate antibody secretion, the predominant function of ASCs. We assayed antibody production by measuring the concentrated of secreted IgM in the culture supernatants using ELISAs. To account for variation in cell numbers between cultures, results were normalized to IgM secretion per cell. Within the ASC gene signature, there were 35 genes that influenced antibody secretion **(** **Figure 4A** **)**. The reduction in IgM secretion after targeting *Irf4*, *Prdm1* and *Sec61a1* is reflective of the block in differentiation, whereas the remaining 32 genes are potential specific regulators of the antibody-secretion process **(** **Supplementary Figures 2C, D** **)**. Genes whose disruption specifically impaired antibody secretion can be segregated into several groups; genes known to be required for antibody secretion (*Xbp1*, *Ell2*), genes involved in protein folding and ERAD (*Calr*, *Dnajb11*, *Edem1*, *Erlec1*, *Srprb*), genes involved in post-translational modifications (*Ddost*, *Dhdds*, *Dpagt1*, *Fut1, Uba5*), and genes with an unknown function or whose function is not obviously linked with antibody secretion (*Bckdk*, *Bet1*, *Cacna1h*, *Dnajc3*, *Enpp1*, *Fcer1g*, *Fkbp2*, *Fkbp11*, *Fndc3a*, *Fos*, *Isg20*, *Qpctl*, *Tmem66*, *Tns3*, *Trabd*, *Tvp23b*, *Yars*, *Zfyve21*).

There are multiple stages in the secretion process that these genes may be either directly or indirectly regulating and several of these genes (*Bckdk, Cacna1h, Ddost, Ell2, Fndc3a*, *Tvp23b*) were selected for validation and a more in-depth investigation of their role in antibody secretion. *Xbp1* and *Plpp5* were included as additional controls. To determine whether these genes were influencing the transcriptional switch from producing the membrane bound form of immunoglobulin produced by B cells to the secretory form expressed in ASCs, we examined the amount of membrane bound IgM present on the plasma membrane of transduced CD138^+^ cells **(** **Figure 4B** **)**. Cells transduced with sgRNAs targeting *Ell2* displayed an increase in membrane bound IgM, which is in line with the known role of Ell2 in promoting the usage of the distal *Igh* polyadenylation site to drive the production of secretory transcripts (37). No other sgRNAs affected the levels of membrane bound IgM, suggesting that these genes regulate processes further along the antibody-secretion pathway. Decreased antibody secretion may also be due to a reduction in the production of IgM protein, therefore, the total IgM production capacity of the CD138^+^ cells was determined by sequential membrane bound and intracellular labelling of IgM with the same antibody **(** **Figure 4C** **)**. As expected, *Xbp1* targeted cells had decreased levels of IgM as *Xbp1*-deficient ASCs are known to have a reduced capacity to upregulate immunoglobulin production (13, 14). No other targeted genes resulted in decreased total IgM levels, suggesting that they do not regulate the protein production capacity of ASCs per se. The proportion of IgM^+^ cells was consistently greater than 90% for all sgRNAs, demonstrating that the reduction in IgM secretion was not due to increased frequencies of isotype switched cells (data not shown). To examine the rate of secretion per cell, naive should have special character for i B cells were stimulated with LPS for 24 hours then transduced with sgRNAs. At 2 days post-transduction, BFP^+^ cells were sorted and returned to culture to recover for 24 hours before transfer to ELIspot plates. The average spot size was reduced for all genes and was comparable to that observed for undifferentiated (CD138^-^) B cells **(** **Figure 4D, E** **)**. Thus, all sgRNAs examined appear to reduce antibody secretion downstream of protein synthesis.

## Discussion

The generation, survival and function of ASCs is critical for an effective adaptive immune response and underpins the protective immunity elicited by all current vaccinations. Previous work has identified a core group of expressed genes that are shared between all ASC subsets (11) and interrogating this signature provided us with the opportunity to identify novel regulators of ASC biology. We developed an arrayed CRISPR/Cas9-mediated screening system which allowed for the identification of factors essential for the differentiation, survival, proliferation and antibody secretion capacity of primary B cells at a very high resolution.

The ASC gene signature consists of genes encoding proteins of diverse functional categories including gene expression and translation, UPR, protein transport, post-translational modifications, metabolism, receptors and signaling pathways (11, 12). In light of this it was surprising that only five genes within the ASC gene signature were essential for the differentiation to CD138^+^ ASCs to occur *in vitro*, including *Sdc1*, the gene that encodes CD138, *Irf4*, *Prdm1*, *Sec61a1* and *Hspa5*. *Irf4* and *Prdm1* are well known regulators of ASC formation and function (8, 9, 38). *Sec61a1* and *Hspa5*, encode proteins important in protein translocation into the ER and ensuring correct protein folding. *Sec61a1* has been implicated in the differentiation and survival of human ASCs as *SEC61A1* haploinsufficiency causes decreased rates of differentiation *in vitro* and decreased plasmablast populations *in vivo* (39). This study identified two families with *SEC61A1* mutations, one with a nonsense mutation resulting in haploinsufficiency (p.E381*) and the other with a point mutation (V85D). Interestingly, neither of these mutations resulted in changes in peripheral B cell populations, however, plasmablast populations were reduced. Furthermore, the mutation of *SEC61A1* in multiple myeloma cell lines results in UPR activation and cell death (40, 41). This suggests that the absence of differentiation observed in *Sec61a1* targeted mouse B cells is likely due to increased cell death during the differentiation process as cells try to increase their rates of antibody synthesis. *Hspa5* encoded Grp78 is a key regulator of the UPR due to its function as an ER chaperone protein (18). Grp78 binds to unfolded or misfolded proteins in the ER lumen to facilitate correct protein binding, however, it also binds to the ER stress sensors, IRE1a, PERK and ATF6, keeping them in an inactive state. In the absence of Grp78, the ER stress sensors activate downstream processes including the UPR, and if left unrestrained will induce cell death. It is likely that this terminal UPR activation is occurring in the *Hspa5* targeted cells resulting in the decrease in differentiation, survival and proliferation observed in this study.

By altering the parameters of our genetic screen, we were also able to identify 10 genes that act as negative regulators of ASC differentiation. This list included four regulators of gene expression, *Bach2*, *Pax5*, *Ets1* and *Pold1*. Bach2 is known to represses the expression of *Prdm1* (21, 42) and *Bach2* ^-/-^ B cells display increased rates of differentiation, as was also evident in our screen results. Pax5 is a master regulator of B cell identity, and its inactivation in mature B cells results in cells reverting to an earlier progenitor stage (43). Although downregulation of *Pax5* expression is one of the earliest stages of the ASC differentiation process (6), and Pax5 represses many ASC genes (44, 45), this process is not essential as differentiation proceeds if *Pax5* cannot be downregulated (46). Furthermore, others have reported that RNAi knockdown of *Pax5* expression in activated B cells did not alter the rate of differentiation (47). In contrast, we observed increased rates of differentiation in the targeted cells, suggesting that Pax5 downregulation, while not essential, may still be a limiting step in normal ASC differentiation. This discrepancy is potentially due to a more complete loss of *Pax5* following CRISPR editing while the residual levels of *Pax5* following RNAi may be sufficient for the differentiation process to occur normally. Ets1 has been shown to negatively regulate ASC differentiation specifically induced by the TLR9 ligand CpG (48, 49), however our data suggests a broader function for Ets1 in controlling the rate of ASC differentiation. Interestingly, Ets1 is proposed to act by maintaining *Pax5* expression and post-translationally inhibiting Blimp-1 (49). In keeping with this gatekeeper function, variants in *ETS1* has been linked to several autoimmune conditions including systemic lupus erythematosus (50) and multiple sclerosis (51). Interestingly, targeting *Pold1* caused an increase in differentiation to a similar extent as these key transcription factors. *Pold1* encodes the catalytic subunit of the DNA polymerase delta (PolD) complex, which is involved in the synthesis of the lagging strand during DNA replication and in several DNA damage repair pathways (23), and its mutation in humans results in immunodeficiency (52). We found that *Pold1* loss resulted in a slowed cell cycle in activated B cells. This coupled with prior reports showing B cells that spend a prolonged time in G1 display dramatically increased rates of differentiation (25, 26) suggest that *Pold* loss indirectly increased to rate of ASC differentiation by slowing the cell cycle.

Many of the genes identified in this screen as being essential for antibody secretion encode components of the ER protein processing pathway. This pathway involves a multitude of processes (targeting to the ER, polypeptide import, folding, *N*-linked glycosylation, recognition of misfolded proteins, and targeting of misfolded proteins for degradation) (18) and hits from this screen have been implicated in almost every stage of this pathway (**Figure 4F**) *Srprb* encodes a component of the signal recognition complex, which is controls the co-translational targeting of polypeptides to the ER (53). *Calr* and *Dnajb11* are involved in maintaining ER homeostasis through their roles as chaperones to promote correct protein folding (54, 55). *Edem1* and *Erlec1* are components of the ERAD pathway (56, 57). *Ddost*, *Dhdds*, *Dpagt1* and *Fut1* are all involved in post-translational modification, with *Ddost*, *Dhdds* and *Dpagt1* being required for the synthesis and attachment of *N*-linked glycosylations and *Fut1* being a factor regulating protein fucosylation (58–61). Correct protein glycosylation is essential for facilitating correct protein folding, preventing protein degradation by the ERAD pathway, trafficking from the ER to the golgi, movement through the golgi and transport to the plasma membrane (62). All of these processes are required for antibody secretion, therefore, targeting genes regulating the addition of glycans is likely affecting at least one of these processes.

Several recent studies have also used a CRISPR-Cas9 screening approach to identify regulators of ASC differentiation (15, 63, 64). Although each group used independently curated gene lists for their boutique sgRNA library, making a direct comparison of the results difficult, a relatively small number of common genes essential for ASC differentiation were identified in each study (*Prdm1*, *Irf4* and *Hspa5*). It is also noteworthy that the prior studies identified glycosylation machinery and components of the ERAD and UPR pathways as being essential for ASC differentiation and/or survival (63, 64), while, with the exception of *Hspa5* and *Sec61a1*, we observed that targeting these pathways specifically disrupted antibody secretion. A potential explanation for this discrepancy is that these previous screens all used the induced germinal center culture system, where the B cells were kept alive for longer (65). The shorter timeframe of our screen may allow for this block in antibody secretion to be detected before the accumulation of protein becomes high enough to trigger the terminal UPR, leading to a selective loss of ASCs. These caveats aside, these studies collectively provide a wealth of new information on genes required for ASCs differentiation and function.

The genes required for antibody secretion represent excellent candidates for the development of new small molecules to treat antibody-mediated diseases including autoimmune conditions, allergy, transplant rejection and the plasma cell malignancy multiple myeloma. It is interesting to note, however, that there are 27 additional genes within the ASC gene signature that are implicated in the UPR that did not have a measurable effect on antibody secretion in this assay (**Figure 4F**). There may be redundancy in this pathway so targeting only one gene at a time may not have any effect on secretion rates. The genes identified by this screen may also highlight potential weak links in the antibody secretion process that may underlie immunodeficiency syndromes. As highlighted above, human *SEC61A1* haploinsufficiency has recently been demonstrated to cause a primary antibody deficiency through impaired ASC differentiation (39), whereas *IRF4* haploinsufficiency has been linked to Whipple’s disease caused by the inability to control infection with the bacteria *Tropheryma whipplei* (66). Mutations in the *BTD* gene cause Biotinidase deficiency, a treatable deficiency in biotin that may have an immunodeficiency component (67, 68). Mutations in several other regulators identified in our screen may also result in antibody immunodeficiency, including *CACNA1H*, a calcium channel linked to epilepsy. Two patients with *CACNA1H* mutations have been reported to show selective antibody deficiency (69), whereas patients harboring mutations in the genes encoding ribosomal proteins have been documented to develop common variable immune deficiency (70). The remaining genes from our screen that are involved in ASC differentiation, proliferation or antibody production represent strong additional candidates for primary antibody deficiency genes.

## Materials and methods

### Mice

Experimental mice were bred and maintained on a C57BL/6 genetic background and housed in the Walter and Eliza Hall Institute (WEHI) animal facility in a specific pathogen free environment. Animal experiments were conducted in accordance with protocols approved by the WEHI animal ethics committee. Rosa26-lox-STOP-lox-Cas9-IRES-GFP mice (71) were bred with B6-Cre-deleter mice to generate the constitutive Cas9 transgenic strain.

### B cell isolation and culture

Naïve splenic B cells were isolated using a B cell isolation kit (Miltenyi Biotech) and cultured in B cell medium (RPMI 1640, 10% FCS, 2 mM L-Glutamine, 1 mM sodium pyruvate, 10 mM HEPES, 50 μM β-mercaptoethanol, 1% non-essential amino acids) supplemented with 10 μg/mL LPS (Sigma-Aldrich) ±10 ng/mL mouse IL-4 (R&D Systems). For proliferation analysis, non-proliferating lymphocytes were separated using a Percoll (GE Healthcare) density gradient prior to B cell isolation and cells were labelled with the division tracking dye, CTY (Invitrogen).

### Flow cytometry

Cells were stained with monoclonal antibodies specific for CD138 (281-2; BioLegend) or IgM (331.12; eBioScience). Intracellular staining was performed using BD Cytofix/Cytoperm (BD Biosciences). Cell viability was determined by the addition of 1 μg/mL Propidium Iodide (PI; Sigma-Aldrich), 1 μg/mL FluoroGold (Sigma-Aldrich) or 1 μL/mL eFluoro-780 (eBioscience).

### Enzyme-linked immunosorbent assay (ELISA)

Plates were coated with anti-IgM (1 μg/mL; Southern Biotech) overnight. Plates were washed with PBS/0.04% Tween-20, PBS, then water before the addition of cell culture supernatant or IgM standard (TEPC183; Sigma-Aldrich) to the appropriate wells. After 4 hours, plates were washed and incubated with anti-IgM-HRP (1 μg/mL; Southern Biotech) for a further 4 hours. Plates were washed and bound IgM was visualized by the addition of 2,2’-Azino-bis(3-ethylbenzothiazoline-6-sulfonic acid) (ABTS; Sigma-Aldrich) substrate solution (0.54 mg/mL ABTS, 10.5 mg/mL citric acid, 15 mg/mL trisodium citrate dihydrate, 0.03% hydrogen peroxide).

### Enzyme-linked immunospot (ELISpot)

Multiscreen HA plates (Millipore) were coated with anti-IgM diluted in 0.2 M carbonate buffer for 4 hours. Plates were washed with PBS before cells in B cell medium were added. Plates were then incubated at 37°C 10% CO_2_ for 14-18 hours. Plates were washed as in the ELISA method before the addition of anti-IgM-HRP. IgM secreting cells were visualized by the addition of 3-amino-9-ethylcarbazole (AEC; Sigma-Aldrich) solution (0.05 M sodium acetate, 0.25 mg/mL AEC, 2% *N,N*,-Dimethyl Formamide, 0.03% hydrogen peroxide).

### Production of lentiviral vectors

Individual sgRNA plasmids were obtained from the Sanger Arrayed Mouse Whole Genome Lentiviral CRISPR Library (Sigma-Aldrich, #MSANGERG) that co-expressed BFP. HEK293T cells were maintained in DMEM/10% FCS and plated 16 hours prior to transfection at a density of 2x10^4^ or 1.5x10^6^ cells for 96-well and 10 cm^2^ plates respectively. pMDL1-gag-pol, pCAG-Eco, pRSV-REV and sgRNA plasmids were combined at a ratio of 3:2:2:3. Fugene6 Transfection Reagent (Promega) was added to the plasmid mix at a ratio of 3 μl FuGENE6 to 1 μg DNA and incubated for 30 minutes before the FuGENE6-DNA mixture was added to HEK293T cultures. Transfected HEK293T cells were BFP^+^ (**Supplementary Figure 1A** **)**. Lentivirus containing supernatant was collected 48 hours post-transfection, and either used fresh or stored at -80 °C.

### Transduction of primary B cells

Non-tissue culture treated 96-well plates were coated with Retronectin (32 μg/mL; produced in house) for 4 hours and plates were blocked with PBS/2% BSA prior to the addition of cells and lentiviral containing supernatant. Each well received only a single sgRNA expressing lentiviral supernatant. Plates were then centrifuged at 1200 rpm for 90 minutes. Following centrifugation, supernatant was removed, and cells were resuspended in B cell medium containing LPS ± IL-4. The rate of transduction (proportion of BFP^+^ cells (**Supplementary Figure 1B** **)** and impact of CRISPR sgRNA on ASC differentiation (proportion of total BFP^+^ cells that are CD138^+^) and cell number was determined by flow cytometry, and the impact on antibody secretion was quantified by ELISA. The proportion of BFP^+^ CD138^+^ ASCs and the antibody secretion rate were compared to untransduced controls for each plate.

### Analysis of publicly available RNAseq data

To generate a list of FoB specific genes, we reanalyzed RNA-sequencing data published by Shi et al. (GSE60927) (11). The count table was downloaded and gene with at least 1 count per million (CPM) in at least three samples were included downstream analysis (72, 73). Count data were normalized using the trimmed mean of M-values (TMM) method, and differential gene expression analysis was performed using the limma-voom pipeline (limma version 3.40.6) (72, 74, 75). FoB specific genes had a 3-fold higher expression in FoB samples compared to all ASC subsets (≤0.05 false discovery rate). Heatmaps of logCPM were generated using pheatmap.

### Statistical analysis

Statistical significance was determined by two-way ANOVA with multiple comparisons.

## Data availability statement

The original contributions presented in the study are included in the article/**Supplementary Materials**, further inquiries can be directed to the corresponding author/s.

## Ethics statement

The animal study was reviewed and approved by Walter and Eliza Hall Institute Animal Ethics Committee.

## Author contributions

Conceptualization, ST and SN. Methodology, ST, IK, and MH. Investigation, ST and IK. Writing – original draft, ST and SN. Writing – review and editing, ST, IK, EH, MH, SW, and SN. Resources, EH, MH, SW, and SN. Supervision, EH, SW and SN. All authors contributed to the article and approved the submitted version.
