# Supplementary material for: An arrayed CRISPR screen of primary B cells reveals the essential elements of the antibody secretion pathway
Source: Front Immunol. 2023 Feb 13;14:1089243. doi: 10.3389/fimmu.2023.1089243 (PMC9969136; doi:10.3389/fimmu.2023.1089243)
Supplement: Supplementary file 1 [file DataSheet_1.docx]

**SUPPLEMENTARY MATERIAL**

**An arrayed CRISPR screen of primary B cells reveals the essential elements of the antibody secretion pathway**

Stephanie Trezise^1-3^, Isabella Y. Kong^1,2,4^, Edwin D. Hawkins^1,2^ Marco J. Herold^1,2^, Simon N. Willis,^1,2^ Stephen L. Nutt^1,2*^

*^1^Walter and Eliza Hall Institute of Medical Research, 1G Royal Parade, Parkville, Victoria, Australia, ^2^Department of Medical Biology, The University of Melbourne, Parkville, Victoria, Australia, ^3^Center for Immunology and Inflammatory Diseases, Massachusetts General Hospital, Harvard Medical School, Harvard University, Boston, MA, ^4^Department of Pediatrics, Division of Pediatric Hematology/Oncology, Weill Cornell Medicine, New York, NY.*

**List of Supplementary Material**

**Supplementary Figures**

Supplementary Figure 1. Optimization and validation of the screening method.

Supplementary Figure 2. Comparison of sgRNA effects in replicate screens.

Supplementary Figure 3. Negative screen gene list and workflow validation.

**Supplementary Tables**

Supplementary Table 1. Genes included in the screen for positive regulators of ASC differentiation and function.

Supplementary Table 2. Genes included in the screen for negative regulators of ASC differentiation

**SUPPLEMENTARY FIGURES**

**
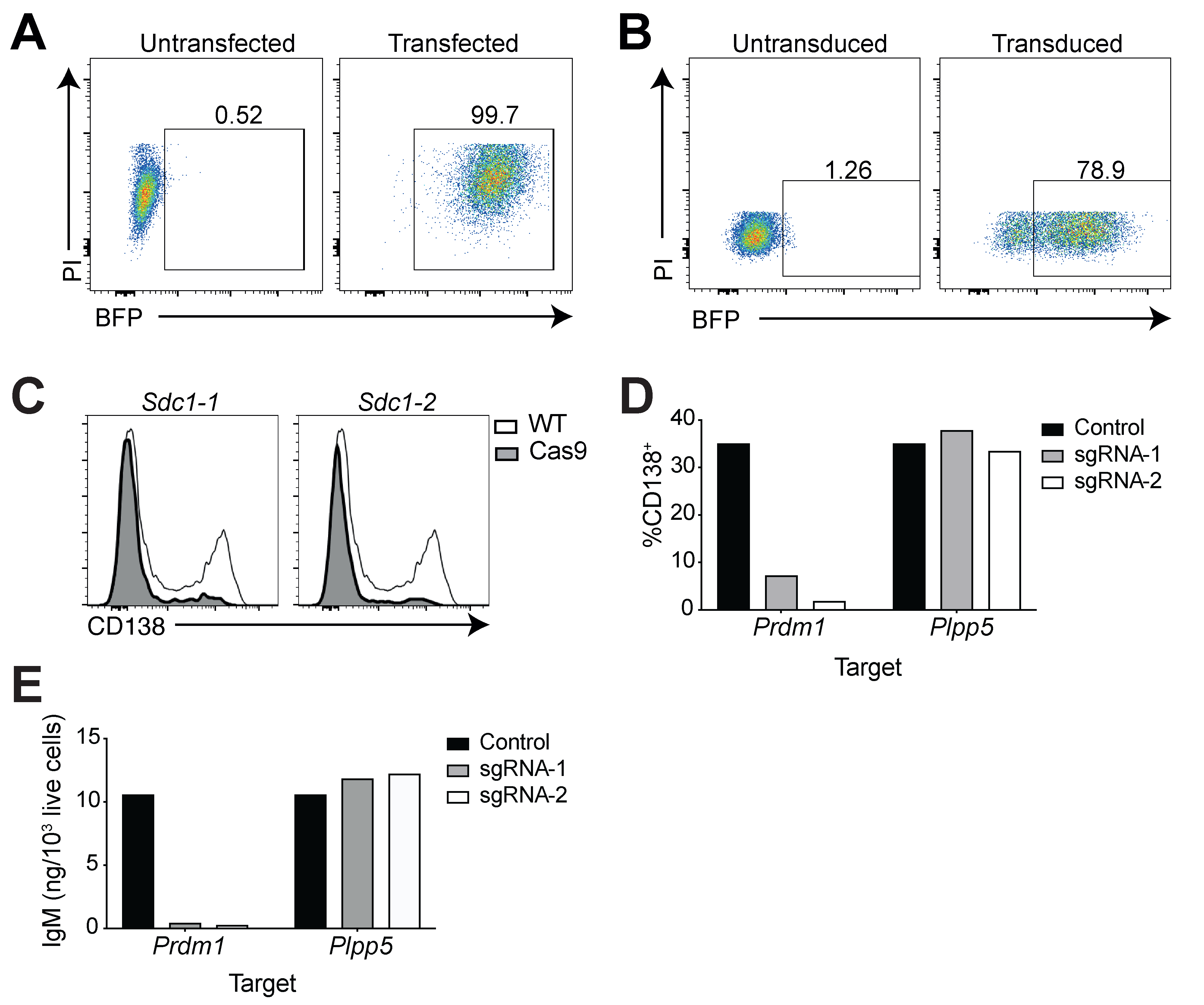
**

**Supplementary Figure 1. Optimization and validation of the screening method.**

(A) Efficiency of transfection of 293T cells in 96-well plates as measured by the proportion of BFP^+^ cells at 48 hours post-transfection. (B) Naïve splenic B cells were activated for 24 hours with LPS before transduction with lentivirus. Following transduction, cells were cultured in LPS for 3 days before transduction efficiency was determined by the proportion of BFP^+^ cells. (C) Naïve splenic B cells from WT or Cas9 transgenic mice were activated with LPS and transduced with sgRNAs targeting *Sdc1*. At 3 days post-transduction, the surface expression of CD138 was assessed by flow cytometry. (D-E) Naïve splenic B cells from Cas9 transgenic mice were activated with LPS and transduced with sgRNAs targeting *Prdm1* or *Plpp5*. At 3 days post-transduction, the proportions of CD138^+^ cells were assessed by flow cytometry (D) and IgM secretion was measured by ELISA (E).

**
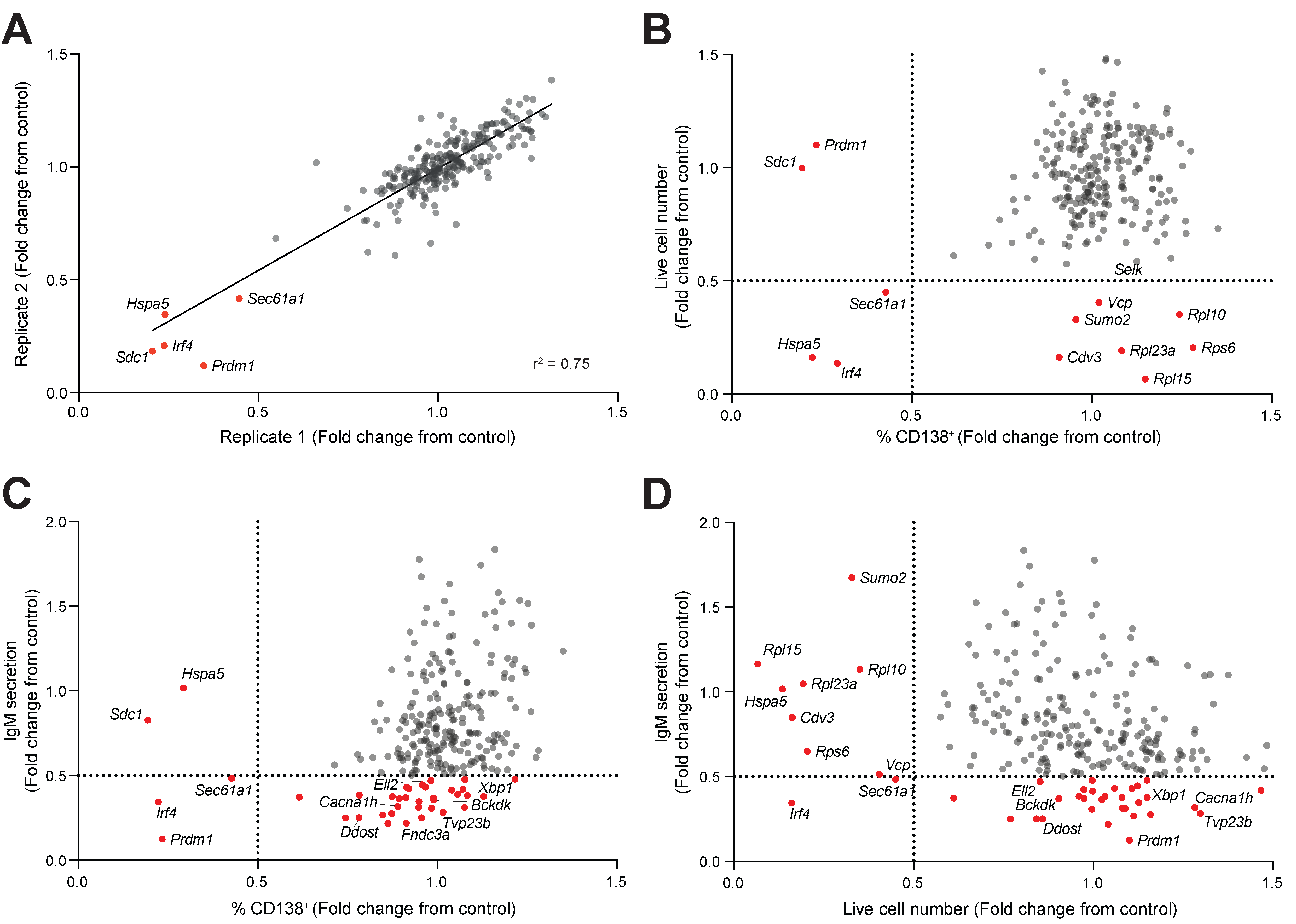
**

**Supplementary Figure 2.** **Comparison of sgRNA effects in replicate screens.**

(A) Workflow as in **Figure 1a**. Data are presented as fold changes in the proportion of CD138^+^ cells for each targeted gene relative to the untransduced control in each screen. Genes with a fold change of 0.5 or lower in both screens are labelled and highlighted in red. Pearson correlation was used to determine r^2^. (B-D) Correlation graphs showing the fold change of each gene for the indicated parameters. Genes with a fold change of greater than 0.5 in any parameter are highlighted in red. (B) Frequency of CD138^+^ cells among transduced population compared to total live cell number. (C) Frequency of CD138^+^ cells among transduced population compared to IgM secretion per cell. (D) Total live cell number compared to IgM secretion per cell.

**
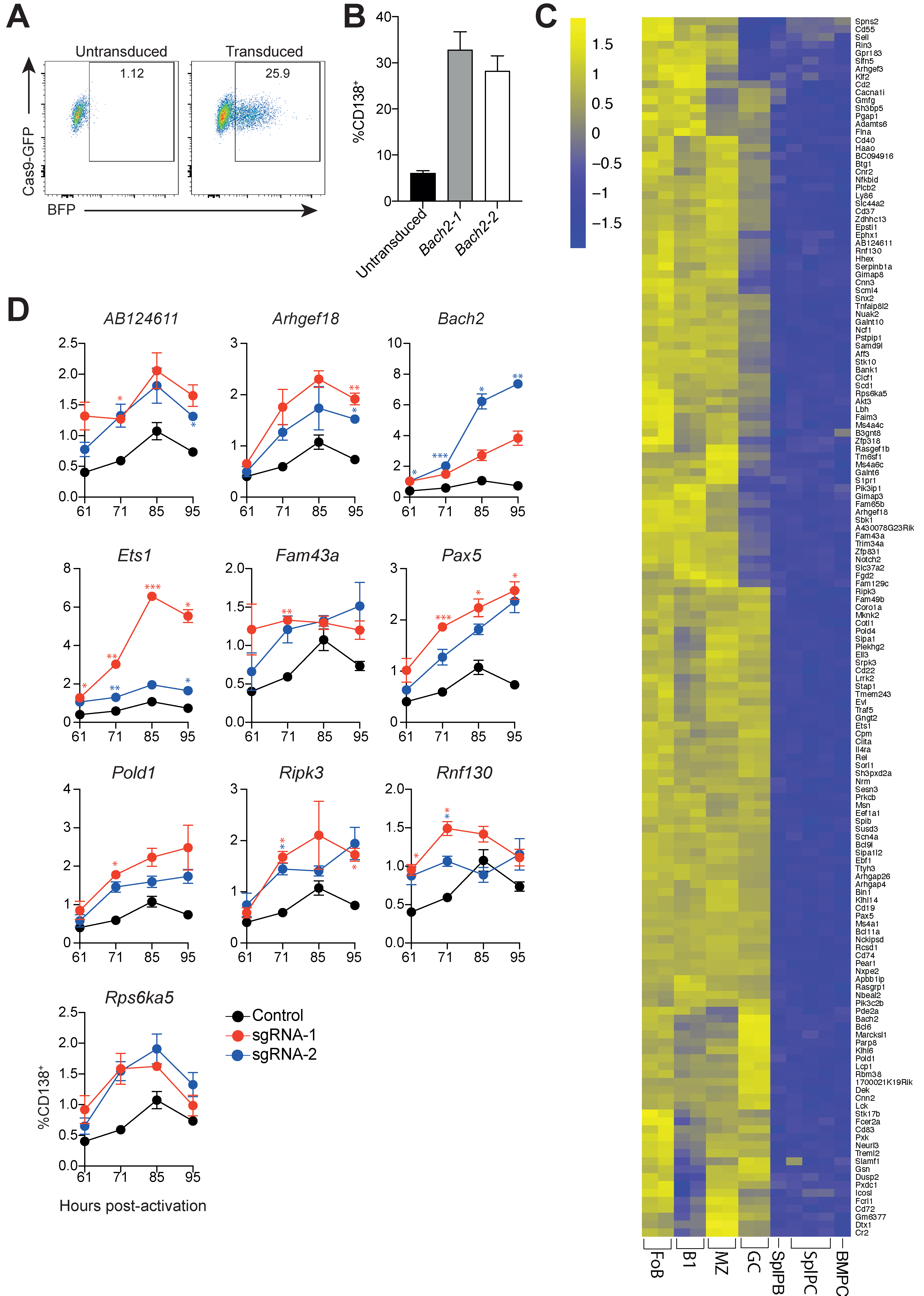
**

**Supplementary Figure 3. Negative screen gene list and workflow validation.**

(A-B) Naïve splenic B cells were isolated from Cas9 transgenic mice, transduced with sgRNAs targeting *Bach2* and activated with LPS+IL-4. The proportion of transduced (BFP^+^) cells was determined on day 2 (A) and the proportion of CD138^+^ cells (within the BFP gate) was determined on day 4 (B) post-transduction by flow cytometry and compared to untransduced controls. (C) Expression profile of 155 genes that are downregulated in ASC populations compared to follicular B cell (FoB) samples (FDR ≤ 0.05, fold-change ≥ 3, FPKM in FoB samples ≥ 32). Heatmap is color-coded according to Z-score. B1, peritoneal B1 cell; MZ, marginal zone B cell; GC, germinal center B cell; SplPB, spleen plasmablast; SplPC, spleen plasma cell; BMPC, bone marrow plasma cell. Data were obtained from Shi *et al*. ^1^ (C) Proportion of CD138^+^ cells in cultures transduced with the indicated sgRNAs at the indicated time post-activation with LPS+IL-4. Data points represent the mean of triplicate wells and error bars indicate the S.E.M. * p<0.05, ** p<0.01, *** p<0.001. Data in C is representative of 2 independent experiments.

**Supplementary Table 1. Genes included in the screen for positive regulators of ASC differentiation and function.**

| **Gene name** | **MGI ID** |
| --- | --- |
| 1700017B05Rik | MGI:1921461 |
| 4632428N05Rik | MGI:1921298 |
| Abcb1b | MGI:97568 |
| Ada | MGI:87916 |
| Adk | MGI:87930 |
| Alad | MGI:96853 |
| Alcam | MGI:1313266 |
| Aldh18a1 | MGI:1888908 |
| Aldh9a1 | MGI:1861622 |
| Alg2 | MGI:1914731 |
| Ap2m1 | MGI:1298405 |
| Arf4 | MGI:99433 |
| Arfgap3 | MGI:1913501 |
| Asns | MGI:1350929 |
| Atat1 | MGI:1913869 |
| Atf5 | MGI:2141857 |
| Atl2 | MGI:1929492 |
| Atp6v0a1 | MGI:103286 |
| BC003331 | MGI:2385108 |
| Bckdk | MGI:1276121 |
| Bet1 | MGI:1343104 |
| Bhlha15 | MGI:891976 |
| Bst2 | MGI:1916800 |
| Btd | MGI:1347001 |
| Cacna1h | MGI:1928842 |
| Calr | MGI:88252 |
| Capn5 | MGI:1100859 |
| Cars | MGI:1351477 |
| Ccdc117 | MGI:2144383 |
| Ccpg1 | MGI:1196419 |
| Cd28 | MGI:88327 |
| Cdv3 | MGI:2448759 |
| Chid1 | MGI:1915288 |
| Chpf | MGI:106576 |
| Chst1 | MGI:1924219 |
| Cited2 | MGI:1306784 |
| Clptm1l | MGI:2442892 |
| Cltb | MGI:1921575 |
| Cnpy2 | MGI:1928477 |
| Cnst | MGI:2445141 |
| Cope | MGI:1891702 |
| Coro2b | MGI:2444283 |
| Cpeb2 | MGI:2442640 |
| Cpox | MGI:104841 |
| Creb3l2 | MGI:2442695 |
| Creg1 | MGI:1344382 |
| Creld2 | MGI:1923987 |
| Ctse | MGI:107361 |
| D17Wsu104e | MGI:2156020 |
| Dap | MGI:1918190 |
| Ddost | MGI:1194508 |
| Dennd5b | MGI:2444273 |
| Derl1 | MGI:1915069 |
| Derl3 | MGI:1917627 |
| Desi1 | MGI:106313 |
| Dhdds | MGI:1914672 |
| Dnajb11 | MGI:1915088 |
| Dnajb9 | MGI:1351618 |
| Dnajc3 | MGI:107373 |
| Dpagt1 | MGI:1196396 |
| Edem1 | MGI:2180139 |
| Edem2 | MGI:1915540 |
| Edem3 | MGI:1914217 |
| Ell2 | MGI:2183438 |
| Endou | MGI:97746 |
| Enpp1 | MGI:97370 |
| Entpd1 | MGI:102805 |
| Epcam | MGI:106653 |
| Ergic1 | MGI:1914708 |
| Erlec1 | MGI:1914003 |
| Erp44 | MGI:1923549 |
| Fam214a | MGI:2387648 |
| Fam46c | MGI:1921895 |
| Fcer1g | MGI:95496 |
| Fkbp11 | MGI:1913370 |
| Fkbp2 | MGI:95542 |
| Fndc3a | MGI:1196463 |
| Fndc3b | MGI:1919257 |
| Fos | MGI:95574 |
| Fut1 | MGI:109375 |
| Fut8 | MGI:1858901 |
| Galnt2 | MGI:894694 |
| Gfpt1 | MGI:95698 |
| Glb1 | MGI:88151 |
| Glipr1 | MGI:1920940 |
| Glt8d1 | MGI:1923735 |
| Gne | MGI:1354951 |
| Gpr155 | MGI:1915776 |
| Gpr55 | MGI:2685064 |
| Gvin1 | MGI:1921808 |
| H13 | MGI:95886 |
| H1f0 | MGI:95893 |
| Hdlbp | MGI:99256 |
| Herpud1 | MGI:1927406 |
| Hid1 | MGI:2445087 |
| Hist1h1c | MGI:1931526 |
| Hsd11b1 | MGI:103562 |
| Hsp90b1 | MGI:98817 |
| Hspa13 | MGI:1309463 |
| Hspa5 | MGI:95835 |
| Htatip2 | MGI:1859271 |
| Hyou1 | MGI:108030 |
| Idh2 | MGI:96414 |
| Ift20 | MGI:1915585 |
| Iqcb1 | MGI:2443764 |
| Irf4 | MGI:1096873 |
| Isg20 | MGI:1928895 |
| Itm2c | MGI:1927594 |
| Kcnn4 | MGI:1277957 |
| Kdelr1 | MGI:1915387 |
| Kdelr2 | MGI:1914163 |
| Krtcap2 | MGI:1913309 |
| Larp1b | MGI:1914604 |
| Leprotl1 | MGI:1915442 |
| Lman1 | MGI:1917611 |
| Lrrc59 | MGI:2138133 |
| Ly6c1 | MGI:96882 |
| Ly6c2 | MGI:3712069 |
| Ly6k | MGI:1923736 |
| Maged1 | MGI:1930187 |
| Magt1 | MGI:1914325 |
| Man1b1 | MGI:2684954 |
| Manea | MGI:2444484 |
| Mars | MGI:1345633 |
| Mcfd2 | MGI:2183439 |
| Mettl9 | MGI:1914862 |
| Mgat2 | MGI:2384966 |
| Mnda | MGI:3041120 |
| Mrp63 | MGI:1915090 |
| Mt1 | MGI:97171 |
| Mt2 | MGI:97172 |
| Mtdh | MGI:1914404 |
| Mthfd2 | MGI:1338850 |
| Mzb1 | MGI:1917066 |
| Nans | MGI:2149820 |
| Nars | MGI:1917473 |
| Ndufa1 | MGI:1929511 |
| Neil1 | MGI:1920024 |
| Nucb1 | MGI:97388 |
| Nudt22 | MGI:1915573 |
| Oosp1 | MGI:2149290 |
| Os9 | MGI:1924301 |
| Oxct1 | MGI:1914291 |
| P4hb | MGI:97464 |
| Pck2 | MGI:1860456 |
| Pdia6 | MGI:1919103 |
| Pon2 | MGI:106687 |
| Pon3 | MGI:106686 |
| Ppapdc1b | MGI:1919160 |
| Pqlc3 | MGI:2444067 |
| Praf2 | MGI:1859607 |
| Prdm1 | MGI:99655 |
| Prdx4 | MGI:1859815 |
| Prg2 | MGI:103294 |
| Prmt7 | MGI:2384879 |
| Prrc1 | MGI:1916106 |
| Pycr1 | MGI:2384795 |
| Qpctl | MGI:1914619 |
| Rapgef3 | MGI:2441741 |
| Reln | MGI:103022 |
| Rexo2 | MGI:1888981 |
| Rgcc | MGI:1913464 |
| Rhob | MGI:107949 |
| Rhobtb1 | MGI:1916538 |
| Rpl10 | MGI:105943 |
| Rpl15 | MGI:1913730 |
| Rpl23a | MGI:3040672 |
| Rpn1 | MGI:98084 |
| Rpn2 | MGI:98085 |
| Rps27l | MGI:1915191 |
| Rps6 | MGI:98159 |
| Sdc1 | MGI:1349162 |
| Sdf2l1 | MGI:2149842 |
| Sec11c | MGI:1913536 |
| Sec22b | MGI:1338759 |
| Sec24a | MGI:1924621 |
| Sec24d | MGI:1916858 |
| Sec61a1 | MGI:1858417 |
| Sec63 | MGI:2155302 |
| Sel1l | MGI:1329016 |
| Selk | MGI:1931466 |
| Selm | MGI:2149786 |
| Sepp1 | MGI:894288 |
| Serinc5 | MGI:2444223 |
| Serpina3f | MGI:2182838 |
| Sil1 | MGI:1932040 |
| Slamf7 | MGI:1922595 |
| Slc12a4 | MGI:1309465 |
| Slc33a1 | MGI:1332247 |
| Slc35b1 | MGI:1343133 |
| Slc39a11 | MGI:1917056 |
| Slc39a4 | MGI:1919277 |
| Slc39a7 | MGI:95909 |
| Slc3a2 | MGI:96955 |
| Slc44a1 | MGI:2140592 |
| Slc7a5 | MGI:1298205 |
| Slpi | MGI:109297 |
| Snd1 | MGI:1929266 |
| Snx9 | MGI:1913866 |
| Spcs1 | MGI:1916269 |
| Spcs2 | MGI:1913874 |
| Spcs3 | MGI:1923937 |
| Spint2 | MGI:1338031 |
| Spon1 | MGI:2385287 |
| Srp54a | MGI:1346087 |
| Srp54c | MGI:3714359 |
| Srpr | MGI:1914648 |
| Srprb | MGI:102964 |
| Ssr1 | MGI:105082 |
| Ssr2 | MGI:1913506 |
| Ssr3 | MGI:1914687 |
| Ssr4 | MGI:1099464 |
| St6gal1 | MGI:108470 |
| Stt3a | MGI:105124 |
| Sumo2 | MGI:2158813 |
| Surf4 | MGI:98445 |
| Tapbpl | MGI:2384853 |
| Tigit | MGI:3642260 |
| Tmed2 | MGI:1929269 |
| Tmed3 | MGI:1913361 |
| Tmed9 | MGI:1914761 |
| Tmem176a | MGI:1913308 |
| Tmem176b | MGI:1916348 |
| Tmem184b | MGI:2445179 |
| Tmem214 | MGI:1916046 |
| Tmem116 | MGI:1924712 |
| Tmem248 | MGI:1918917 |
| Tmem258 | MGI:1916288 |
| Tmem39a | MGI:1915096 |
| Tmem66 | MGI:1915137 |
| Tnfrsf17 | MGI:1343050 |
| Tns3 | MGI:2443012 |
| Top1 | MGI:98788 |
| Tpst1 | MGI:1298231 |
| Trabd | MGI:1915226 |
| Tram2 | MGI:1924817 |
| Trp53inp1 | MGI:1926609 |
| Tvp23b | MGI:1914760 |
| Txndc11 | MGI:1923620 |
| Txndc5 | MGI:2145316 |
| Uba5 | MGI:1913913 |
| Ubb | MGI:98888 |
| Ube2j1 | MGI:1926245 |
| Ubxn4 | MGI:1915062 |
| Ufc1 | MGI:1913405 |
| Uso1 | MGI:1929095 |
| Vcp | MGI:99919 |
| Vimp | MGI:95994 |
| Wbp5 | MGI:109567 |
| Wipi1 | MGI:1261864 |
| Xbp1 | MGI:98970 |
| Yars | MGI:2147627 |
| Yipf2 | MGI:1922016 |
| Yipf5 | MGI:1914430 |
| Yipf6 | MGI:1925179 |
| Zbp1 | MGI:1927449 |
| Zfp280b | MGI:1927865 |
| Zfyve21 | MGI:1915770 |

**Supplementary Table 2. Genes included in the screen for negative regulators of ASC differentiation.**

| **Gene name** | **MGI ID** |
| --- | --- |
| 1700021K19Rik | MGI:1915160 |
| A430078G23Rik | MGI:2442135 |
| AB124611 | MGI:3043001 |
| Adamts6 | MGI:1347348 |
| Aff3 | MGI:106927 |
| Akt3 | MGI:1345147 |
| Apbb1ip | MGI:1861354 |
| Arhgap26 | MGI:1918552 |
| Arhgap4 | MGI:2159577 |
| Arhgef18 | MGI:2142567 |
| Arhgef3 | MGI:1918954 |
| B3gnt8 | MGI:2385269 |
| Bach2 | MGI:894679 |
| Bank1 | MGI:2442120 |
| BC094916 | MGI:3584522 |
| Bcl11a | MGI:106190 |
| Bcl6 | MGI:107187 |
| Bcl9l | MGI:1933114 |
| Bin1 | MGI:108092 |
| Btg1 | MGI:88215 |
| Cacna1i | MGI:2178051 |
| Cd19 | MGI:88319 |
| Cd2 | MGI:88320 |
| Cd22 | MGI:88322 |
| Cd37 | MGI:88330 |
| Cd40 | MGI:88336 |
| Cd55 | MGI:104850 |
| Cd72 | MGI:88345 |
| Cd74 | MGI:96534 |
| Cd83 | MGI:1328316 |
| Ciita | MGI:108445 |
| Clcf1 | MGI:1930088 |
| Cnn2 | MGI:105093 |
| Cnn3 | MGI:1919244 |
| Cnr2 | MGI:104650 |
| Coro1a | MGI:1345961 |
| Cotl1 | MGI:1919292 |
| Cpm | MGI:1917824 |
| Cr2 | MGI:88489 |
| Dek | MGI:1926209 |
| Dtx1 | MGI:1352744 |
| Dusp2 | MGI:101911 |
| Ebf1 | MGI:95275 |
| Eef1a1 | MGI:1096881 |
| Ell3 | MGI:2673679 |
| Ephx1 | MGI:95405 |
| Epsti1 | MGI:1915168 |
| Ets1 | MGI:95455 |
| Evl | MGI:1194884 |
| Faim3 | MGI:1916419 |
| Fam129c | MGI:3686743 |
| Fam43a | MGI:2676309 |
| Fam49b | MGI:1923520 |
| Fam65b | MGI:2444879 |
| Fcer2a | MGI:95497 |
| Fcrl1 | MGI:2442862 |
| Fgd2 | MGI:1347084 |
| Flna | MGI:95556 |
| Galnt10 | MGI:1890480 |
| Galnt6 | MGI:1891640 |
| Gimap3 | MGI:1932723 |
| Gimap8 | MGI:2685303 |
| Gm6377 | MGI:3647255 |
| Gmfg | MGI:1927135 |
| Gngt2 | MGI:893584 |
| Gpr183 | MGI:2442034 |
| Gsn | MGI:95851 |
| Haao | MGI:1349444 |
| Hhex | MGI:96086 |
| Icosl | MGI:1354701 |
| Il4ra | MGI:105367 |
| Klf2 | MGI:1342772 |
| Klhl14 | MGI:1921249 |
| Klhl6 | MGI:2686922 |
| Lbh | MGI:1925139 |
| Lck | MGI:96756 |
| Lcp1 | MGI:104808 |
| Lrrk2 | MGI:1913975 |
| Ly86 | MGI:1321404 |
| March1 | MGI:1920175 |
| Marcksl1 | MGI:97143 |
| Mknk2 | MGI:894279 |
| Ms4a1 | MGI:88321 |
| Ms4a4c | MGI:1927656 |
| Ms4a6c | MGI:2385644 |
| Msn | MGI:97167 |
| Nbeal2 | MGI:2448554 |
| Ncf1 | MGI:97283 |
| Nckipsd | MGI:1931834 |
| Neurl3 | MGI:2429944 |
| Nfkbid | MGI:3041243 |
| Notch2 | MGI:97364 |
| Nrm | MGI:2146855 |
| Nuak2 | MGI:1921387 |
| Nxpe2 | MGI:1925502 |
| Parp8 | MGI:1098713 |
| Pax5 | MGI:97489 |
| Pde2a | MGI:2446107 |
| Pear1 | MGI:1920432 |
| Pgap1 | MGI:2443342 |
| Pik3c2b | MGI:2685045 |
| Pik3ip1 | MGI:1917016 |
| Plcb2 | MGI:107465 |
| Plekhg2 | MGI:2141874 |
| Pold1 | MGI:97741 |
| Pold4 | MGI:1916995 |
| Prkcb | MGI:97596 |
| Pstpip1 | MGI:1321396 |
| Pxdc1 | MGI:1914145 |
| Pxk | MGI:1289230 |
| Rasgef1b | MGI:2443755 |
| Rasgrp1 | MGI:1314635 |
| Rbm38 | MGI:1889294 |
| Rcsd1 | MGI:2676394 |
| Rel | MGI:97897 |
| Rin3 | MGI:2385708 |
| Ripk3 | MGI:2154952 |
| Rnf130 | MGI:1891717 |
| Rps6ka5 | MGI:1920336 |
| S1pr1 | MGI:1096355 |
| Samd9l | MGI:1343184 |
| Sbk1 | MGI:2135937 |
| Scd1 | MGI:98239 |
| Scml4 | MGI:2446140 |
| Scn4a | MGI:98250 |
| Sell | MGI:98279 |
| Serpinb1a | MGI:1913472 |
| Sesn3 | MGI:1922997 |
| Sh3bp5 | MGI:1344391 |
| Sh3pxd2a | MGI:1298393 |
| Sipa1 | MGI:107576 |
| Sipa1l2 | MGI:2676970 |
| Slamf1 | MGI:1351314 |
| Slc37a2 | MGI:1929693 |
| Slc44a2 | MGI:1915932 |
| Slfn5 | MGI:1329004 |
| Snx2 | MGI:1915054 |
| Sorl1 | MGI:1202296 |
| Spib | MGI:892986 |
| Spns2 | MGI:2384936 |
| Srpk3 | MGI:1891338 |
| Stap1 | MGI:1926193 |
| Stk10 | MGI:1099439 |
| Stk17b | MGI:2138162 |
| Susd3 | MGI:1913579 |
| Tm6sf1 | MGI:1933209 |
| Tmem243 | MGI:3606159 |
| Tnfaip8l2 | MGI:1917019 |
| Traf5 | MGI:107548 |
| Treml2 | MGI:2147038 |
| Trim34a | MGI:2137359 |
| Ttyh3 | MGI:1925589 |
| Zdhhc13 | MGI:1919227 |
| Zfp318 | MGI:1889348 |
| Zfp831 | MGI:3641861 |
